# Determinants of the Use of Breast Cancer Screening Among Women Workers in Urban Mexico

**Published:** 2008-03-15

**Authors:** Kristin Marie Wall, Georgina Mayela Núñez-Rocha, Ana María Salinas-Martínez, Sergio R Sánchez-Peña

**Affiliations:** School of Public Health, University of Texas Health Science Center. Ms Wall also is affiliated with the Unidad de Investigación Epidemiológica y en Servicios de Salud, Instituto Mexicano del Seguro Social, Monterrey, Nuevo León, México; Unidad de Investigación Epidemiológica y en Servicios de Salud, Instituto Mexicano del Seguro Social, Monterrey, Nuevo León, México; Unidad de Investigación Epidemiológica y en Servicios de Salud, Instituto Mexicano del Seguro Social, Monterrey, Nuevo León, México; Departamento de Parasitología, Universidad Autónoma Agraria Antonio Narro, Saltillo, Coahuila, México

## Abstract

**Introduction:**

This case-control study aimed to determine critical factors influencing the use of clinical breast examination and mammography among women workers in Monterrey, Mexico.

**Methods:**

We determined case and control status from survey results. Cases were defined in accordance with the guidelines of the Official Mexican Standard as lack of at least one clinical breast examination during the past year by surveyed women. For women older than 40 years, cases were further defined as lack of at least one mammogram in the previous 2 years and, for women older than 50, lack of a mammogram in the previous year. Controls were defined as adherence by surveyed women to these guidelines. Participants (N = 306 clerks aged 18–60) provided information about their practices, knowledge, and perceptions regarding breast cancer screening. Factors identified by odds ratio analysis as significantly different between cases and controls were analyzed by multivariate logistic regression.

**Results:**

Survey participants' knowledge about the utility of breast self-examination (odds ratio, 6.0; 95% confidence interval, 1.0–33.9), perception that the health care system has enough equipment and personnel for clinical breast examination (odds ratio, 4.7; 95% confidence interval, 1.7–13.2), and perception that they have enough time to wait for and receive clinical breast examinations (odds ratio, 2.5; 95% confidence interval, 1.1–5.8) significantly predisposed women to use screening services independent of years of formal education, number of pregnancies, number of living children, hours worked per week, and monthly family income.

**Conclusion:**

Perception of organizational and structural factors played a significant role in screening use. Our findings have implications for the general population, provider practices, community interventions, and future development of strategies to increase use of screening services in similar locales.

## Introduction

Two decades ago, breast cancer was not a major health concern in the developing world (1). However, recent demographic and epidemiologic changes led to increased breast cancer incidence in areas where health care systems still lack the early detection programs and treatment services to effectively combat this disease (2). Consequently, rates of death from breast cancer are highest in economically disadvantaged countries, largely because of late-stage diagnosis (3).

With the goal of formulating recommendations for early detection of breast cancer in developing countries, the 2002 Global Summit Consensus Conference on International Breast Health Care focused on several key issues, including educating and empowering women to adhere to guidelines for breast screening, developing infrastructure for diagnosing and treating breast cancer, and educating primary health care professionals. The 2002 Global Summit also identified social and cultural variables that may be barriers to use of breast cancer screening services, including beliefs that cancer is invariably fatal, spouse/partner lack of acceptance of screening, fear of lack of social support, and preference for traditional medicine (4).

Mexico exemplifies a country caught in this demographic and epidemiologic transition. With socioeconomic development, urbanization, and increased entrance of women into the labor market have come trends linked to increased breast cancer incidence, including obesity, sedentary lifestyle, late parity, nulliparity, and oral contraceptive use (5,6). Breast cancer is second only to cervical-uterine cancer as a leading cause of cancer-related death among Mexican women. In 2005, the death rate was nearly 16 per 100,000 women (7). Despite public health services that offer breast self-examination (BSE) training to women and free clinical breast examination (CBE) (8), the Mexican Institute of Oncology reported that 80% to 85% of breast cancers are detected in advanced stages (9).

Monterrey, the capital of the state of Nuevo León, is one of many urban areas where chronic diseases such as diabetes, heart disease, and cancer are leading causes of morbidity and mortality (10). Monterrey is highly representative of the demographic transition, with a relatively elevated socioeconomic level and a high percentage of women in the workforce (11). In 2005, the rate of death from breast cancer in Nuevo León was 20.6 per 100,000 women — well above national levels (7) — and use of screening services in Monterrey is deficient by national standards (10). Exploring the cancer screening practices and perceptions of this population is therefore critical to determining factors related to use of CBE and mammography.

The Health Belief Model describes and predicts preventive actions related to cancer by focusing on interactions among health behaviors, practices, and use of services (12). The model determines health behavior by perceptions and values and by demographic, sociocultural, structural, and organizational factors described earlier (13). Consistent with this model, we hypothesized that use of breast cancer screening services among women workers is influenced by sociodemographic and sociocultural factors, in addition to organizational and structural factors related to the health care system. No studies have investigated these determinants among Mexican women formally employed in the labor market, a population more likely to have risk behaviors associated with breast cancer, such as elevated socioeconomic level, nulliparity, and late age at first childbirth (5).

Therefore, we identified and quantified critical factors related to use of breast cancer screening services among women workers in Monterrey, generating novel results for understanding patterns of advanced-stage diagnosis and for modeling strategies to increase early breast cancer detection.

## Methods

### Study design and sample

This case-control study included formally employed female store clerks working and residing in Monterrey, Mexico. Clerks were selected on the basis of their ability to be surveyed in their place of work away from family and peer influences that could interfere with accurate questionnaire response. The majority of women surveyed (94%) were the only workers present at the place of business; when more than one female worker was present and eligible to be surveyed, we emphasized the importance of each woman responding individually.

Women aged 18 years or older were eligible to participate because BSE and CBE are recommended for women starting at this age, and screening at young ages offers an opportunity for women and their doctors or nurses to discuss changes in their breasts, methods of early detection, and factors in the woman's history that might predict future breast cancer. We excluded women with a history of cancer. A minimum sample size of 149 cases and 149 controls was established for 95% confidence in the results and 80% power to detect an odds ratio (OR) of 2.

We determined case-control status from survey results. Cases were defined in accordance with the Official Mexican Standard (*Norma Oficial Mexicana*, 041-SSA2-2002) as lack of at least one CBE during the previous year. For women older than 40, we further defined cases as lack of at least one mammogram in the previous 2 years and, for women older than 50, in the previous year. We defined controls as adherence to the Official Mexican Standard guidelines by surveyed women (14). The 324 women who completed surveys were categorized as cases (171) or controls (153) and age-matched in a 1:1 ratio within 5-year age-group distributions. We excluded 18 cases that remained unmatched, giving a final sample size of 306 (153 cases and 153 controls). The Institutional Review Board of the Mexican Social Security Institute (*Instituto Mexicano del Seguro Social*) in Monterrey approved study methodology and protocol measures.

### Survey administration

Before administration, surveys — written in basic Spanish to be easily understood by the general population — were validated with a small sample of working women (N = 15) and revised to increase participant comprehension and compliance. The first author (KMW) visited 312 randomly selected businesses throughout Monterrey, accounting for districts of various sizes, and distributed one-third of surveys during each of the three work shifts. Six businesses did not have at least one eligible female clerk on shift. In businesses with eligible participants, the first author explained the purpose of the study and survey, including its anonymous and voluntary nature. The first author left surveys with participants in their place of work and collected them later the same day at a time requested by the participant, usually within 1 hour, to allow for adequate response time during shift breaks or lulls. At collection, respondents were asked if they needed clarification on any of the questions.

Of the 379 women asked to participate, 330 (87.1%) agreed and gave informed consent, and 49 (12.9%) declined. Those who declined most frequently cited lack of time (92%), followed by the belief that cancer is invariably fatal, therefore negating the utility of the survey (8%). We excluded the six (1.8%) participants who did not complete at least 80% of the survey.

### Survey measures

The survey asked about sociodemographic, sociocultural, educational, and organizational and structural factors related to the health care system. Sociodemographic variables included respondent's age, age at first childbirth, number of pregnancies and living children, hours worked per week, and monthly family income. Other variables determined the repeat breast cancer screening practices of the respondents, including the number of BSEs in the previous year and lifetime number of CBEs and mammograms. Response types were 1) open-ended, 2) categorical yes/no, or 3) ranked on a 5-point Likert scale.

Sociocultural variables were defined as beliefs and values ingrained in a culture. Respondents provided answers on a 5-point Likert scale (from "very little" to "very much") to the following questions: Are you afraid/feel embarrassed to have your breast examined/receive a mammography from health care personnel? Does your spouse/partner accept that health care personnel examine your breasts/perform a mammography? Do you believe cancer is always fatal?

Educational factors included years of formal education and knowledge about breast cancer screening utility and guidelines. Respondents answered the questions: Do you feel BSE/CBE/mammography is important for good health (5-point Likert scale ranging from "marginally" to "extremely" reduced to categorical accurate vs inaccurate)? Do you know how often you should examine your breasts (weekly, monthly, every 3 months, yearly; reduced to categorical accurate vs inaccurate)? Do you know the screening recommendations for CBE/mammography (weekly, monthly, every 3 months, yearly; reduced to categorical accurate vs inaccurate)?

Organizational and structural factors related to the health care system are built into medical encounters that may influence use of services, such as availability of heath resources and quality of care. The survey focused on the respondent's subjective point of view, given that perceptions can be barriers to screening regardless of the objective state of the health care system. Respondents answered the following questions: Did you feel health care personnel and equipment were sufficient for providing CBE/mammography tests (5-point Likert scale ranging from "marginally" to "extremely" reduced to categorical yes vs no)? Did you feel the quality of service provided for CBE/mammography testing was sufficient (5-point Likert scale ranging from "marginally" to "extremely" reduced to categorical yes vs no)? Other structural factors related to the health care system encompass an individual's resources and opportunities to obtain medical attention, such as costs and waiting times. All formally employed persons in Mexico are provided government-funded health insurance covering CBE and mammography screening services, thus removing financial problems as a barrier to screening. Respondents answered how they felt about the waiting time for obtaining CBE and mammography the last time they solicited such services and the waiting time for receiving examinations.

### Statistical analyses

We created two education-related indices, each comprising three variables. The first assessed participant knowledge of BSE, CBE, and mammography utility ("screening utility index"). This index coded for accurate versus inaccurate knowledge of the utility of all three detection methods. However, because of an unacceptable level of internal consistency (Cronbach α = 0.48), items could not be combined and were retained in the analysis as separate variables. The second index evaluated participant knowledge of BSE, CBE, and mammography screening guidelines ("screening guidelines index"). This index coded for accurate versus inaccurate knowledge of screening guidelines for all three detection methods. This index reached an acceptable level of internal consistency (Cronbach α = 0.75) and was retained in the analysis along with the individual variables comprising the index.

Survey data were entered directly into SPSS 10.0 for Windows (1999) (SPSS, Inc, Chicago, Illinois). Sociodemographic data and quantitative information concerning cancer screening practices were analyzed by descriptive statistics and *t *tests. Univariate OR analysis evaluated the strength of association between participants' knowledge and misperceptions of breast cancer screening and service use. Factors identified as significantly different between cases and controls were entered into multivariate logistic regression models.

## Results

### Sociodemographic characteristics and repeat screening practices

Significantly more control women than case women had a formal education of high school or more, had at least one pregnancy and at least one living child, and worked 40 hours per week or more (Table 1). Significantly more control women than case women engaged in repeat cancer screening practices (number of BSEs in the previous year and lifetime CBEs and mammograms).

### Univariate analysis

Variables most strongly associated with use of CBE and mammography were related to educational factors (Table 2). Control women were more likely to have accurate knowledge of the utility of BSE, CBE, and mammography and their respective screening guidelines. Perceptions about structural and organizational barriers also were strongly associated with use of breast cancer screening services.

Four survey variables (data not shown) were not significantly associated with case-control status as determined by OR analysis. These were the belief that cancer is not invariably fatal (OR, 0.7; 95% confidence interval [CI], 0.5–1.1), spouse/partner acceptance of CBE (OR, 3.1; 95% CI, 0.5–17.2), spouse/partner acceptance of mammography (OR, 1.6; 95% CI, 0.5–4.6), and perceptions of reasonable waiting time to receive results of mammography (OR, 2.8; 95% CI, 0.7–12.2).

### Multivariate logistic regression analysis

Variables identified as significantly associated with screening use were entered into multivariate logistic regression analysis (Table 3). Participants' knowledge about the utility of BSE, their perception that the health care system has enough medical personnel and equipment available for a CBE or mammogram, and their perception that the length of time they have to wait for a CBE or mammogram was acceptable were significantly associated with use of the screening services, independent of years of formal education, number of pregnancies, number of living children, hours worked per week, or monthly family income (McFadden's pseudo *R* ^2^ = 0.292).

## Discussion

Our study directly linked the use of breast cancer screening services with years of formal education and accurate knowledge of early detection guidelines. A principal factor for use of screening services was knowledge about BSE screening guidelines, a well-documented indicator in studies of Hispanic women (15-17). Our study found additional determinants of CBE and mammography use: women's perception that 1) the health care system has enough equipment and personnel to perform these procedures and 2) the time they had to wait to receive these procedures was acceptable.

Survey participants who perceived that enough equipment and personnel were available for CBE were more inclined to adhere to national screening guidelines for both CBE and mammography. Previous studies indicate the importance of these organizational factors to use of preventive services (18). Indeed, they may facilitate or preclude use of breast cancer screening regardless of service adequacy or accessibility (19).

Social change and epidemiologic transition, especially regarding changes in fertility and breast cancer incidence and awareness in Mexico, also may influence perceptions of the accessibility and quality of medical care concerning sufficient equipment and personnel for CBE. As educational and workforce opportunities improve for women, health awareness and the consequent demand for quality health services increase (1). However, inadequacies in facilities and staff training may undermine these demands (20). Coupled with the high frequency with which women first seek medical care for advanced-stage cancer, which requires aggressive therapy and increases patient suffering, organizational inadequacies can propagate beliefs that available medical equipment and personnel cannot adequately treat or cure breast cancer (21).

In addition, participants who perceive they have enough time to wait for and obtain an annual CBE were more inclined to adhere to both CBE and mammography in accordance with national screening guidelines. Structural barriers, such as lack of time, have been described in relation to breast cancer screening, especially the inability of employed women to take work leave or pay child care expenses during an absence (22). This perception also may result from low breast health priority. When not at work, women commonly care for children or other family members, and priorities for their limited time may not include personal health concerns, especially preventive ones such as BSE, CBE, and mammography. A prevailing emphasis on curative care often amplifies low prioritization of breast health (23).

Finally, we investigated the influence of fear and embarrassment with regard to CBE and mammography, spouse/partner acceptance of CBE and mammography, and cancer fatality beliefs. Although the sociocultural profile of a population must be considered if a cancer-detection program is to be effective (24) − and several studies document the importance of these factors among Hispanic women (15,17) − we did not find these perceptions to be significantly associated with use of screening services.

Our study is subject to information bias because of participants' potential inability to quantitatively recall screening practices and the inaccurate disclosure of personal information such as sexual and reproductive history. To minimize this bias, we surveyed participants away from family and peer influence, assured them of response anonymity, and gave them enough time to carefully consider and answer all questions. Because of these considerations and the high participant response rate, we believe our data are generalizable and have reliably determined critical factors related to use of the breast cancer screening service by this population.

We based our study on the assumption that misperceptions can be barriers to screening, regardless of the objective state of the health care system. Therefore, we aimed to determine how users and potential users perceived this system. For example, perceptions of inadequacies in equipment and personnel for CBE may represent actual deficiencies of the system or may result from misconceptions by the general population. The actual state of the breast cancer screening program must be evaluated in terms of these perceptions to determine whether community interventions to increase breast cancer education, screening awareness, and breast health priority are enough or whether macro-level policy interventions aimed at organizational reform of the current system also are necessary.

Accurate information and perceptions of the availability of sufficient resources (i.e., personnel, equipment, and time) most strongly determined use of breast cancer screening services. Hypothesized sociocultural factors did not play a significant role. Our findings describing the lack of use of screening services by women workers in Monterrey have implications for the general population, provider practices, community interventions, and future development of strategies to increase use of screening services in similar locales.

## Figures and Tables

**Table 1:** Sociodemographic Profile and Repeat Breast Cancer Screening Practices Among Women Workers in Monterrey, Mexico, 2006

Variable	Case^a^ (n = 153)	Control^b^ (n = 153)	*P* Value
**Age group, y **			
18–39	80.4%	79.7%	.40
≥40	19.6%	20.3%	
**Formal education**			
Low (less than high school)	60.9%	31.8%	<.0001
High (high school or more)	39.1%	68.2%	
**Pregnancies**			
Yes	56.9%	79.7%	.005
No	43.1%	20.3%	
**Living children**			
Yes	52.9%	78.4%	.003
No	47.1%	21.6%	
**Hours worked per week**			
≤39	31.4%	20.3%	.009
≥40	68.6%	79.7%	
**Breast self-examination in previous year**			
Yes	34.6%	95.4%	<.0001
No	65.4%	4.6%	
**Clinical breast examination in lifetime**			
Yes	26.8%	93.5%	<.0001
No	73.2%	6.5%	
**Mammograms in lifetime^c^**			
Yes	30.0%	93.5%	<.0001
No	70.0%	6.5%	

a A case was defined as lack of at least one clinical breast examination during the previous year by surveyed women. For women older than 40, a case was further defined as lack of at least one mammogram in the previous 2 years and, for women older than 50, in the previous year.

b A control was defined as adherence by surveyed women to the above guidelines.

c Among women aged 40 or older (31 controls, 30 cases).

**Table 2:** Knowledge and Perceptions About Use of Breast Cancer Screening Services Among Women Workers in Monterrey, Mexico, 2006

Factor	Case^a^ (n = 153) No. (%)	Control^b^ (n = 153) No. (%)	OR (95% CI)^c^
**Accurate knowledge (educational factors)**			
Utility of BSE	87 (56.9)	151 (98.7)	57.3 (13.7–239.7)
Utility of CBE	133 (86.9)	150 (98.0)	7.5 (2.2–25.9)
Utility of mammography	128 (83.7)	145 (94.8)	3.5 (1.5–8.1)
BSE screening guidelines	45 (29.4)	119 (77.8)	8.4 (5.0–14.0)
CBE screening guidelines	41 (26.8)	143 (93.5)	39.1 (18.8–81.4)
Mammography screening guidelines	29 (19.0)	97 (63.4)	7.4 (4.4–12.5)
Screening guidelines index	18 (11.8)	109 (71.2)	18.6 (10.1–34.0)
**Sociocultural factor**s			
CBE: No fear or embarrassment	112 (73.2)	142 (92.8)	4.7 (2.3–9.6)
Mammography: No fear or embarrassment	116 (75.8)	135 (88.2)	2.4 (1.3–4.4)
**Perceptions of medical care: organizational and structural factors**			
CBE: Enough time to wait for and obtain	35 (22.9)	70 (45.8)	6.2 (3.7–10.2)
Mammography: Enough time to wait for and obtain	30 (19.6)	68 (44.4)	3.3 (2.0–5.5)
CBE: Enough equipment and personnel^d^	31 (62.0)	141 (92.2)	7.2 (3.2–16.4)
Mammography: Enough equipment and personnel^e^	14 (46.7)	24 (77.4)	3.9 (1.3–11.8)
CBE: Quality of attention^d^	34 (68.0)	48 (31.4)	13.9 (4.8–40.7)
Mammography: Quality of attention^e^	14 (46.7)	24 (77.4)	3.9 (1.3– 11.8)

OR indicates odds ratio; CI, confidence interval; BSE, breast self-examination; CBE, clinical breast examination.

a A case was defined as lack of at least one CBE during the previous year by surveyed women. For women older than 40, a case was further defined as lack of at least one mammogram in the previous 2 years and, for women older than 50, in the previous year.

b A control was defined as adherence by surveyed women to the above guidelines.

c *P* < .001.

d Among women who received at least one CBE in their lifetime (153 controls, 50 cases).

e Among women aged 40 or older (31 controls, 30 cases).

**Table 3:** Results of Multivariate Logistic Regression Analysis of Factors Associated With Use of Breast Cancer Screening Services Among Women Workers in Monterrey, Mexico, 2006

Factor	Adjusted OR (95% CI)^a^	*P* Value
Accurate knowledge of utility of BSE	6.0 (1.0–33.9)	.04
Perception that the health care system has enough equipment and personnel for CBE	4.7 (1.7–13.2)	.003
Perception that women have enough time to obtain CBE	2.5 (1.1–5.8)	.03

OR indicates odds ratio; CI, confidence interval; BSE, breast self-examination; CBE, clinical breast examination.

a OR for each variable adjusted for years of formal education, number of pregnancies, number of living children, hours worked per week, and monthly family income.

## References

[1] Robles SC, Galanis E (2002). Breast cancer in Latin America and the Caribbean. Rev Panam Salud Publica.

[2] National Cancer (2002). National Cancer Control Programmes: Policies and managerial guidelines, 2002.

[3] Parkin DM, Bray F, Ferlay J, Pisani P (2005). Global cancer statistics, 2002. CA Cancer J Clin.

[4] Anderson BO, Braun S, Lim S, Smith RA, Taplin S, Thomas DB (2003). Early detection of breast cancer in countries with limited resources. Breast J.

[5] Tovar-Guzmán V, Hernández-Girón C, Lazcano-Ponce E, Romieu I, Hernández Avila M (2000). Breast cancer in Mexican women: an epidemiological study with cervical cancer control. Rev Saude Publica.

[6] Parkin DM, Bray FI, Devesa SS (2001). Cancer burden in the year 2000. The global picture. Eur J Cancer.

[7] (2002). Strategic information from the federal organization. Health: México 2001-2005.

[8] (1992). Main Directorate of Maternal-Infant Attention and Preventive Medicine. National Program of Prevention and Control of Cervicouterine and Breast Cancer.

[9] Alcántara-Rodríguez V, Alonso de Ruiz P, Bosh X, Buiatti E, Cisneros-Arce T, Dehesa-Violante M (1995). [Workshop regarding the link between epidemiological investigation and cancer prevention and control programs]. Salud Pública Mex.

[10] Garza-Elizondo MF, Villarreal-Rios E, Salinas-Martinez AM, Nunez-Rocha GM (2004). [Preventive practices of the residents over 25 years of age in Monterrey and its metropolitan area (Mexico)]. Rev Esp Salud Publica.

[11] La Botz D (1999). Women in Mexican society, the workforce, and the labor movement. Mexican Labor News and Analysis.

[12] Sheeran P, Cooner M, Norman P (2001). Can the theory of planned behavior explain patterns of health behavior change?. Health Psychol.

[13] Glanz K, Rimer BK, Lewis FM (2002). Health behavior and health education: theory, research and practice.

[14] (2003). [NOM 041-SSA2-2002, for the prevention, diagnosis, treatment, control and epidemiological surveillance of breast cancer]. Diario Oficial de la Federación.

[15] Fernandez MA, Tortolero-Luna G, Gold RS (1998). Mammography and Pap test screening among low-income foreign-born Hispanic women in USA. Cad Saude Publica.

[16] Zambrana RE, Breen N, Fox SA, Gutierrez-Mohamed ML (1999). Use of cancer screening practices by Hispanic women: analyses by subgroup. Prev Med.

[17] Ramirez AG, Suarez L, Laufman L, Barroso C, Chalela P (2000). Hispanic women's breast and cervical cancer knowledge, attitudes, and screening behaviors. Am J Health Promot.

[18] Agurto I, Bishop A, Sanchez G, Betancourt Z, Robles S (2004). Perceived barriers and benefits to cervical cancer screening in Latin America. Prev Med.

[19] Aubel J, Rabei H, Mukhtar M (1991). Health workers' attitudes can create communication barriers. World Health Forum.

[20] Anderson BO, Yip CH, Ramsey SD, Bengoa R, Braun S, Fitch M (2006). Breast cancer in limited-resource countries: healthcare systems and public policy. Breast J.

[21] Remennick LI (1999). Preventive behavior among recent immigrants: Russian-speaking women and cancer screening in Israel. Soc Sci Med.

[22] Facione NC, Dodd MJ, Holzemer W, Meleis AI (1997). Helpseeking for self-discovered breast symptoms: implications for early detection. Cancer Pract.

[23] Remennick L (2001). "All my life is one big nursing home." Russian immigrant women in Israel speak about double caregiver stress. Womens Stud Int Forum.

[24] Remennick L (2006). The challenge of early breast cancer detection among immigrant and minority women in multicultural societies. Breast J.

